# Adipogenic Stimulation and Pyrrolidine Dithiocarbamate Induced Osteogenic Inhibition of Dental Pulp Stem Cells Is Countered by Cordycepin

**DOI:** 10.3390/jpm11090915

**Published:** 2021-09-14

**Authors:** Shankargouda Patil, Rodolfo Reda, Nezar Boreak, Hasan Ahmad Taher, Abdulaziz Abu Melha, Ashraf Albrakati, Thilla Sekar Vinothkumar, Mohammed Mustafa, Ali Robaian, Riyadh Alroomy, Rawabi Jaber Ahmed Kharaf, Taif Sharafuddin Kameli, Ahmed Alkahtani, Hosam Ali Baeshen, Vikrant R. Patil, Luca Testarelli

**Affiliations:** 1 Department of Maxillofacial Surgery and Diagnostic Sciences, Division of Oral Pathology, College of Dentistry, Jazan University, Jazan 45142, Saudi Arabia; 2 Department of Oral and Maxillo Facial Sciences, University of Rome La Sapienza, 00161 Rome, Italy; rodolforeda17@gmail.com (R.R.); luca.testarelli@uniroma1.it (L.T.); 3 Department of Restorative Dental Sciences, College of Dentistry, Jazan University, Jazan 45142, Saudi Arabia; nezarboreak@gmail.com (N.B.); vinothkumar_ts@yahoo.com (T.S.V.); 4 Prince Mohammed bin Abdulaziz Hospital, Al Madinah 14214, Saudi Arabia; Hasan-ahmad38@hotmail.com; 5 Department of Restorative Dental Science, College of Dentistry, King Khalid University, Abha 61421, Saudi Arabia; aabumelha@kku.edu.sa; 6 Department of Human Anatomy, College of Medicine, Taif University, P.O. Box 11099, Taif 21944, Saudi Arabia; a.albrakati@tu.edu.sa; 7 Department of Conservative Dental Sciences, College of Dentistry, Prince Sattam bin Abdulaziz University, Al-Kharj 11942, Saudi Arabia; ma.mustafa@psau.edu.sa (M.M.); ali.alQahtani@psau.edu.sa (A.R.); 8 Department of Restorative Dental Sciences, College of Dentistry, Majmaah University, Al Majmaah 11952, Saudi Arabia; r.alroomy@mu.edu.sa; 9 College of Dentistry, Jazan University, Jazan 45142, Saudi Arabia; rawabijaber220@gmail.com (R.J.A.K.); Tf-kamili@hotmail.com (T.S.K.); 10 Department of Restorative Dental Sciences, College of Dentistry, King Saud University, Riyadh 11451, Saudi Arabia; ahkahtani@ksu.edu.sa; 11 Department of Orthodontics, College of Dentistry, King Abdulaziz University, Jeddah 21589, Saudi Arabia; drbaeshen@me.com; 12 Biogenre Private Limited, Pune 412105, India; patilvikrant.r@gmail.com

**Keywords:** cordycepin, dental pulp stem cells, osteogenesis, regeneration

## Abstract

Background: dental pulp-derived stem cells are easy to access and collect and are an excellent source of stem cells for regenerative therapy. These cells can interact with many biomolecules and scaffolds and can pass on the instructive signals to the sites of regeneration where they are used. In this regard cordycepin, a potential biomolecule derived from medicinal mushrooms with a spectrum of bioactive properties such as antioxidant, anti-inflammatory, and anticancer has not yet been tested for its effect on human dental pulp stem cells. Objective: the objective of the present study was to assess the in vitro adipogenic and osteogenic differentiation potential of human dental pulp stem cells with or without induction after administration of cordycepin. Materials and methods: human dental pulp stem cells DPSCs were isolated from a healthy permanent tooth extracted for orthodontic purposes after obtaining informed consent. Flow cytometry technique was used to assess the surface markers of these cells such as CD73, CD90, and CD105, CD34, CD45, and HLA-DR. Further, an MTT assay was performed on the cells after subjecting them to various concentrations of cordycepin. Following this, the adipogenic and osteogenic potential of the dental pulp stem cells was assessed with or without induction under the influence/absence of 5 µM of cordycepin. The results obtained were statistically analyzed and documented. Results: it was found that the dental pulp stem cells showed strong positive expression for CD73, CD90, and CD105 and faint expression of CD34, CD45, and HLA-DR. MTT assay revealed that 5 µM was the optimum concentration of cordycepin for all the assays. Concerning adipogenesis experiments, there was a statistically significant lowering of all the 4 adipogenesis-related genes PPARγ, FABP4, LPL, and C/EBPα following cordycepin treatment in the presence of induction compared to the only induction group and untreated control cells (*p* < 0.05). In connection with osteogenesis, was found that there was a statistically significant increase in the expression of RUNX2, COL1A1, OSX and OCN genes along with the increase in alkaline phosphatase and alizarin red staining in the DPSC treated with cordycepin along with the presence of induction and simultaneous addition of PDTC compared to the control untreated cells and cells treated with induction and simultaneous addition of PDTC (*p* < 0.05). Conclusion: cordycepin can be exploited for its osteopromotive properties and can be used as a bioactive molecule alongside the administration of dental pulp stem cells in the area of regenerative biology and medicine.

## 1. Introduction

Stem cells are a capable means to mankind, can differentiate into an array of cell types, and can be potentially used in the field of regenerative medicine to reconstruct lost tissues and organ parts [1,2,3]. There are loci or niches in the human body which are populated by stem cells. The success of stem cell harvesting largely depends on the access to the collection site and the invasiveness of the collection method. In this regard, the dental pulp is an easy to access site for stem cell collection, and the methodology of the collection is associated with a very low degree of morbidity and invasiveness [4]. It has been demonstrated that dental pulp stem cells (DPSC) are multipotent and can easily be differentiated into bone-forming osteoblasts. It is also noteworthy that DPSC can interact well with scaffolds and biomaterials making the differentiation process easy and well-orchestrated [4].

Regarding the use of stem cells for regeneration of bone tissues, it should be understood that the bone is a tissue that remains in a state of flux. Bone metabolism is characterized by the interplay between osteoblasts that synthesize bone versus the osteoclasts that resorb bone. It has been understood that bone resorption is a feature of inflammatory conditions such as osteoporosis and osteoarthritis wherein the equilibrium between bone formation and resorption is lost causing disability and functional derangement [5,6]. It has been demonstrated that pro-inflammatory cytokines such as TNF alpha and IL-6 are involved in stimulating osteoclastogenesis and are found in higher amounts in the above-mentioned bone pathoses [6,7]. It is in these challenging clinical conditions that DPSC may be harvested and used to reverse bone loss and herald bone repair. In this regard, it would be worthwhile using naturally derived biomolecules along with the DPSC that could favor bone repair.

It is with this connection that cordycepin or 3′-deoxyadenosine nucleoside adenosine, a bioactive component of cordyceps mushrooms needs to be investigated. Several biological roles including attenuation of oxidative stress, microbial and carcinogenic events, and inflammation have been bestowed on cordycepin [8,9,10,11]. Its ability to counter oxidative stress has allowed its application in protecting the nervous, endocrine, and respiratory systems. Several studies have also reported the beneficial action of cordycepin on inflammatory bone diseases. Cordycepin attenuates TNF-α induced inhibition of adipose-derived mesenchymal stem cell osteogenic differentiation [12]. However, the effect of cordycepin on DPSC has not been investigated so far. Hence, the present study was performed to assess the effects of cordycepin on osteogenic and adipogenic differentiation of DPSC to understand if this naturally derived biomolecule could be used to modulate DPSC in clinical in vivo studies attempting bone regeneration.

## 2. Materials and Methods

### 2.1. Cell Culture Protocol for Human DPSCs

DPSCs were isolated from a healthy permanent tooth extracted for orthodontic purposes after obtaining informed consent. The institutional ethical approval (Ref.no.CODJU-19710) was obtained from the College of Dentistry, Jazan University. Human teeth (premolars) were taken from healthy adults between the ages of 14 and 28 who practiced good oral care. Prior to extraction, the patients were given a chlorhexidine mouth rinse to help decrease oral bacteria. The pulp was extracted using a bur chuck type aerator hand piece in sterile conditions, then carefully removed with sterile forceps, immersed in tubes containing PBS with double strength antibiotic-antimycotic solution, and promptly sent to the laboratory. The explant culture approach reported earlier by Patil et al., 2018, was used to isolate and characterize DPSCs. Pulp tissue was shredded into tiny bits and placed in 35 mm polystyrene plastic culture dishes, to summarize. The tissues were soaked in enough fetal Bovine Serum (FBS) (Gibco, Rockville, MD, USA) to completely cover them. For explant tissue including FBS, a 24 h incubation at 37 °C and 5% CO_2_ was performed; the entire culture system was then maintained in DMEM (Invitrogen, Carlsbad, CA, USA) supplemented with 20% FBS and antibiotic-antimycotic solution at the same temperature and CO_2_ conditions. The culture medium was replaced twice weekly, and an inverted phase-contrast microscope was used to examine cell growth, health, and morphology. Cells were detached at 70–80% confluence and moved to a larger 25-cm^2^ polystyrene culture flask using 0.25 percent trypsin-EDTA solution (Invitrogen, Carlsbad, CA, USA) (Nunc, Rochester, NY, USA). Confluent cells were removed using a 0.25 percent trypsin-EDTA solution and then passaged in continuously for expansion and additional tests. In the experiment, cells from passages 2 to 4 were employed.

### 2.2. Characterization of DPSCs Using Flow Cytometry

Confluent DPSCs were collected with trypsinization and washed twice with PBS for flow cytometry analysis. Anti-human-CD73-APC, anti-human-CD90-APC, anti-human-CD105-APC, anti-human-CD34-PE, anti-human-CD45-FITC, and anti-human-HLA-DR-APC antibodies were then added to the cells and incubated for 30 min at 4 °C (Miltenyi Biotec, Auburn, CA, USA). Antibody-stained cells were washed twice in PBS before being counted at 10,000 cells per sample on an Attune NxT Flow Cytometer (Thermo Fisher Scientific, Waltham, MA, USA). The detection and differentiation of positive and negative signals were accomplished using isotype control.

### 2.3. 3-(4,5-dimethylthiazol-2-yl)-2,5-diphenyltetrazolium Bromide (MTT) Assay of DPSC Following Cordycepin Treatment

The cells were seeded in 96-well plates (1 × 10^4^ cells per well) and given different concentrations of cordycepin (Cpn) (Sigma Aldrich, St. Louis, MO, USA) (0.5 μM, 1 μM, 2.5 μM, 5 μM, 10 μM, 25 μM, and 50 μM). The MTT assay was used to assess cordycepin’s cytotoxicity in DPSCs [13]. The cells were seeded into 96-well plates and grown in the appropriate medium for 48 h. MTT solution (Sig-ma-Aldrich Corp., St. Louis, MO, USA) at a concentration of 0.5 mg/mL was applied to each well after the plates had been incubated for 4 h at 37 °C. The medium was then withdrawn, and 100 μL dimethyl sulfoxide (DMSO) (Sigma-Aldrich Corp., St. Louis, MO, USA) was added to each well. A Multiskan Spectrum spectrophotometer (Thermo Scientific, San Jose, CA, USA) was used to detect the absorbance at 570 nm. 

### 2.4. Adipogenic Differentiation of DPSC under the Influence of Cordycepin

The cells were seeded into a 24-well plate with complete growth media (Nunc, Rochester, NY, USA). After 24 h, the cells were switched to adipogenic conditions (DMEM supplemented with 10% FBS, 1 mM dexamethasone, 10 mM insulin, 200 mM indomethacin, and 0.5 mM isobutyl-methylxanthine (Sigma-Aldrich Corp., St. Louis, MO, USA) twice a week for three weeks. Control, induction, and 5 μM cordycepin treatment with induction were constructed as experimental groups. Fixation of the differentiated adipocytes was conducted using 4% paraformaldehyde. Confirmation was done by the 0.3% oil red O for oil droplets for 1 h.

### 2.5. Osteogenic Differentiation of DPSC under the Influence of Cordycepin (Functional Staining with Alizarin Red S and Alkaline Phosphatase Activity)

A 24-well plate (Nunc, Rochester, NY, USA) with complete growth media was employed with a cell density of 2500 cells/cm^2^. The whole growth media was changed with osteogenic induction medium, which consisted of DMEM with 1% antibiotic-antimycotic, 0.1 M dexamethasone, 50 M ascorbate-2-phosphate, and 10 mM β-glycerophosphate after 24 h (Sigma-Aldrich Corp., St. Louis, MO, USA). There were four experimental groups formed; control group without any treatment, induction group subjected to induction medium only, 100 μM Pyrrolidine dithiocarbamate (PDTC) (a selective NF-κB inhibitor) (Abcam, Cambridge, UK) treatment for 2 h before induction, and 100 μM PDTC (2 h) + 5 µM cordycepin treatment with induction medium. Two times a week, the medium was replaced with a new induction media of the same composition. The cells were fixed with 4% paraformaldehyde and 2% alizarin red S for 21 days to examine differentiation towards osteogenic lineage (pH 4.1–4.3) The staining process took 20 min. The stained cells were dissolved using 4% NaOH and quantitation of alizarin red S-stained osteoblasts was done using a spectrophotometer at 450 nm. 

The alkaline phosphatase activity was examined enzymologically on day 21 for all the experimental groups. After washing with PBS, the cells were incubated in 0.1 M sodium nitrate-sodium carbonate buffer (pH 10.0) containing 1% Triton ×100 (Sigma, St. Louis, MO, USA) and 2 mM magnesium sulfate. Subsequently, 6 mM P-nitrophenyl phosphate was added as the substrate to each well and incubated for 30 min at 37 °C. Finally, 1.5 M sodium hydroxide was added to stop the enzyme-substrate reaction. The absorbance was read spectrophotometrically at 405 nm.

### 2.6. Real-Time Quantitative PCR for Analysis of Gene Expression

The GeneJET RNA purification kit (Thermo Scientific, Vilnius, Lithuania) was used to extract total RNA from the cells. RNA (2 g) was reverse transcribed according to the manufacturer’s instructions using a cDNA synthesis kit (High Capacity, Applied Biosystems, Carlsbad, CA, USA). For each gene, 100 ng cDNA was utilized in a total reaction volume of 20 g. On a Real-Time PCR system (QS5, Applied Biosystems, Foster City, CA, USA), quantitative analysis of genes of interest was performed using the SYBR Green PCR master mix (Applied Biosystems, Austin, TX, USA). The expression of target genes linked to stemness, pluripotency, and differentiation was measured and standardized using the Ct technique to beta-actin as a reference gene. Table 1 shows the list of genes and primers (IDT, Coralville, IA, USA).

## 3. Results

**Figure 1:**
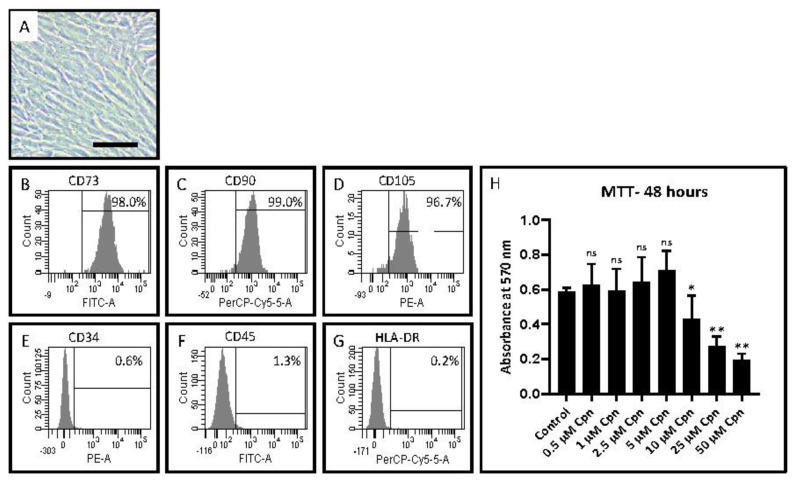
Mesenchymal stem cell (MSC) markers and the MTT assay were used to characterize DPSCs. (**A**) Passage 2 photomicrograph of DPSCs. Scale bar = 100 μm; (**B**–**G**) MSC-specific positive markers CD73, CD90, and CD105, as well as MSC-specific negative markers CD34, CD45, and HLA-DR, were examined in DPSCs; (**H**) For 48 h, DPSCs were treated with various concentrations of cordycepin (0.5 μM, 1 μM, 2.5 μM, 5 μM, 10 μM, 25 μM, and 50 μM) and a comparative study was performed to determine the cytotoxic effect of cordycepin to DPSCs. Cpn: cordycepin. * *p* < 0.05, ** *p* < 0.01.

As mentioned earlier, the DPSC used in the present study were obtained from an academic setup laboratory. However, we performed a flow cytometry protocol to assess stem cell markers expressed by the cell line. It was found that the cells showed strong positive expression for CD 73, CD 90, and CD 105 and faint expression of CD 34, CD 45, and HLA-DR (Table 2, Figure 1A–G).

After stem cell characterization, an MTT assay was performed on the DPSC with various concentrations of cordycepin. Data based on the assay revealed that 5 µM cordycepin was an ideal concentration to carry out further studies (data presented in Table 3 and Figure 1H).

**Figure 2:**
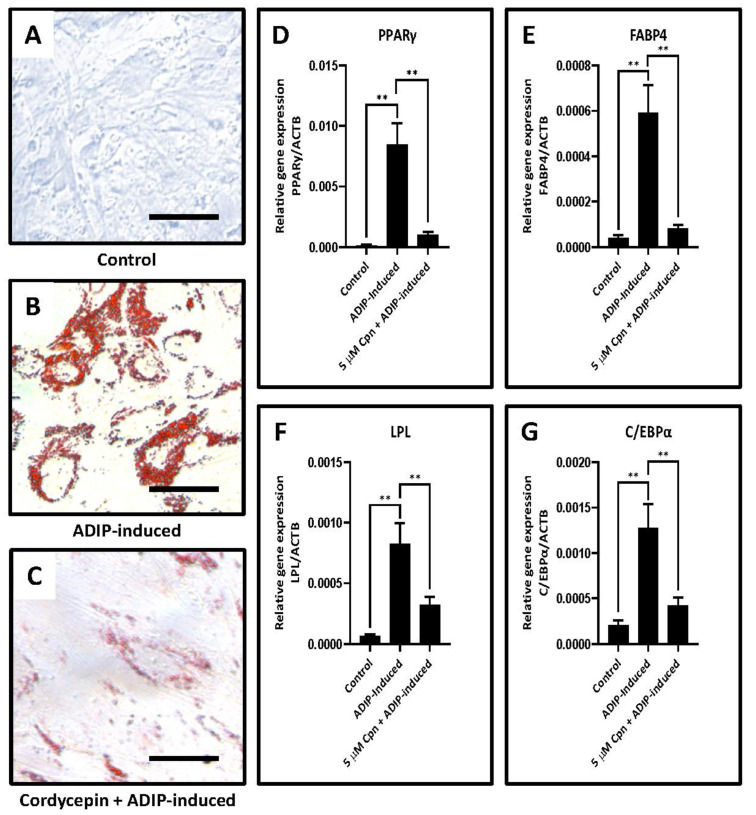
Adipogenic differentiation of DPSCs and quantitative RT-qPCR study of gene expression. (**A**–**C**) Adipogenic differentiation was induced in DPSCs with and without cordycepin administration, and oil red O was used for functional staining. Scale bar = 100 μm; (**D**–**G**) Comparative gene expression study of genes involved in adipogenesis PPARγ, FABP4, LPL, C/EBPα in with and without cordycepin treated adipogenesis induced DPSCs. ns not significant, ** *p* < 0.01. ADIP-induced: adipogenic induction, Cpn: cordycepin, PPARγ: peroxisome proliferator-activated receptor-gamma, FABP4: fatty acid-binding protein 4, LPL: lipoprotein lipase, C/EBPα: CCAAT/enhancer-binding protein alpha.

Concerning the experiments assessing adipogenesis related genes in the DPSC following induction and cordycepin treatment, it was found that there was a statistically significant lowering of all four adipogenesis related genes PPARγ, FABP4, LPL, and C/EBPα following cordycepin treatment in the presence of induction compared to the only induction group and untreated control cells (*p* < 0.05) (data presented in Table 4 and Table 5 and Figure 2D–G). The cell culture images are presented in Figure 2A–C.

**Figure 3:**
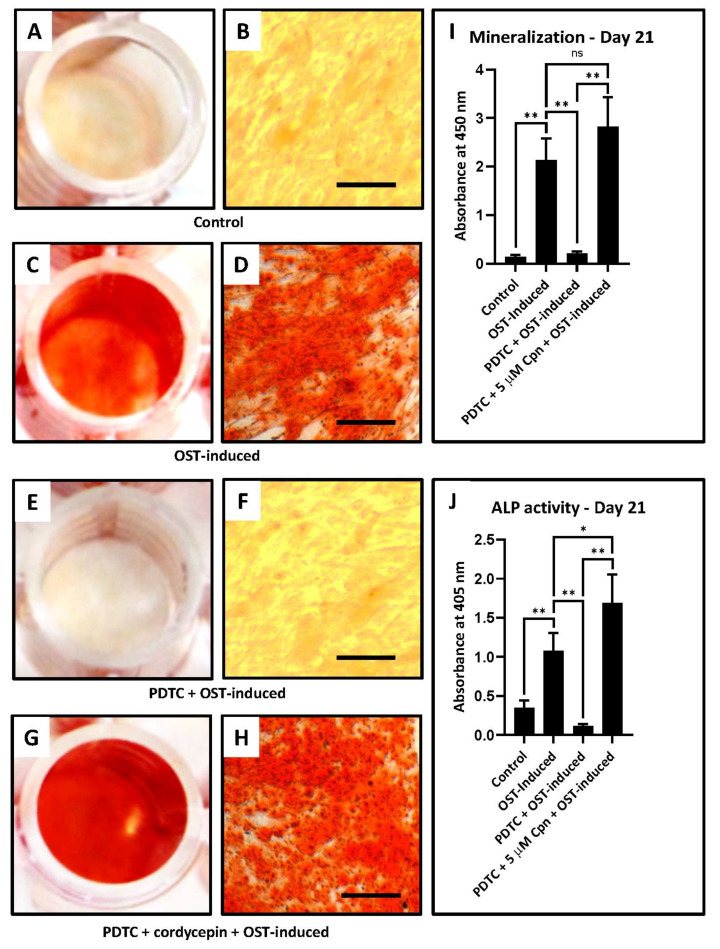
Osteogenic differentiation of DPSCs, quantification of mineralization, and alkaline phosphatase activity. (**A**–**H**) Osteogenic differentiation was induced in DPSCs with and without treatment with cordycepin and inhibition of osteogenesis was induced with PDTC. Functional staining was done with alizarin red S. Scale bar = 100 μm; (I) Comparative quantification of mineralization with and without cordycepin treated osteogenesis induced and osteogenesis inhibited DPSCs; (**J**) Comparative alkaline phosphatase activity with and without cordycepin treated osteogenesis induced and osteogenesis inhibited DPSCs. ns not significant, * *p* < 0.05, ** *p* < 0.01. OST-induced: osteogenic induction, Cpn: cordycepin.

In connection with the ethe DPSC following induction with and without cordycepin treated experiments assessing the osteogenesis related genes and bone-forming characteristics of nt versus control cells along with the use of PDTC a nuclear factor (NF) kappa B inhibitor, it was found that there was a statistically significant increase in the expression of Runt-related transcription factor 2 (RUNX2), collagen, type I, alpha 1 (COL1A1), osterix (OSX) and osteocalcin (OCN) in the DPSC treated with cordycepin along with the presence of induction and simultaneous addition of PDTC compared to the control untreated cells and cells treated with induction and simultaneous addition of PDTC (*p* < 0.05) (Figure 3A–D, Table 6).

**Figure 4:**
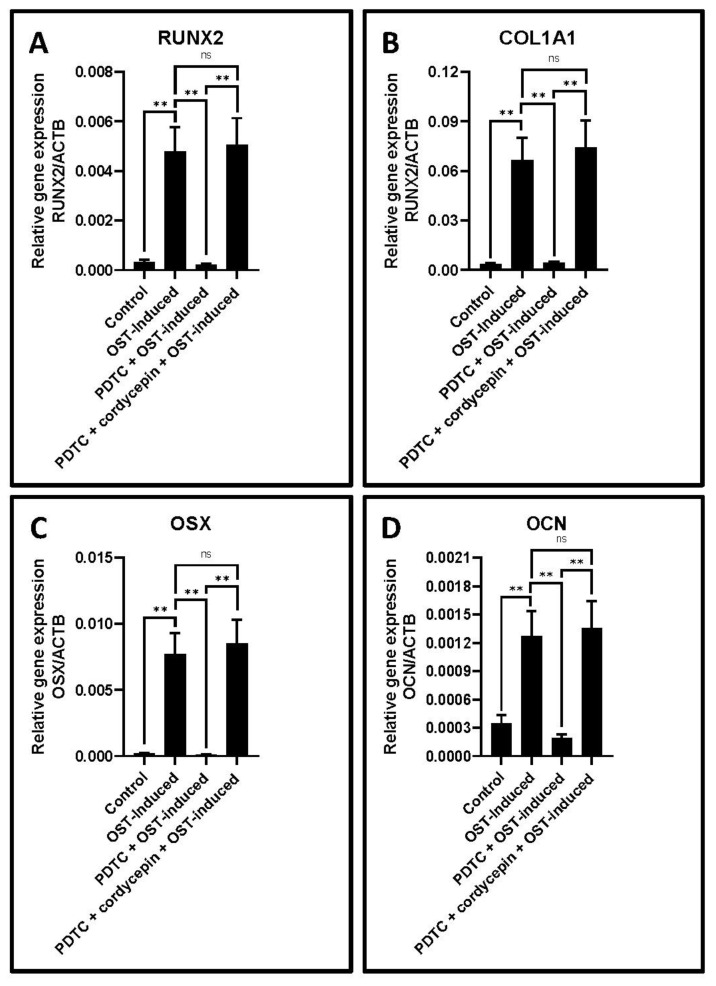
Osteogenic differentiation of DPSCs and quantitative RT-qPCR study of gene expression. (**A**–**D**) Comparative gene expression analysis of osteogenesis-related genes RUNX2, COL1A1, OSX, and OCN in with and without cordycepin treated osteogenesis induced and osteogenesis inhibited DPSCs. ns not significant, ** *p* < 0.01. OST-induced: osteogenic induction, Cpn: cordycepin, RUNX2: Runt-related transcription factor 2, COL1A1: collagen, type I, alpha 1, OSX: transcription factor Sp7/Osterix, OCN: bone gamma-carboxyglutamic acid-containing protein/osteocalcin.

However, there was no significant difference observed in the gene expression between the DPSC treated with cordycepin along with the presence of induction and simultaneous addition of PDTC compared with the induction-only group (*p* > 0.05). Consequently, the bone formation was assessed in the cultures by determining ALP activity and alizarin red staining. In this regard, it was found that the same trend was observed as in the gene expression analysis. Maximum significant alkaline phosphatase (ALP) activity was observed in the DPSC treated with cordycepin along with the presence of induction and simultaneous addition of PDTC compared to the control untreated cells, cells treated with induction and simultaneous addition of PDTC, and the induction only group (*p* < 0.05) (Figure 4A–J, Table 7). 

Concerning the alizarin staining for mineralization assessment, it was found that maximum activity was observed in the DPSC treated with cordycepin along with the presence of induction and simultaneous addition of PDTC compared to the control untreated cells, cells treated with induction, and simultaneous addition of PDTC (*p* < 0.05). However, there was no significant difference observed in the staining intensity between the DPSC treated with cordycepin along with the presence of induction and simultaneous addition of PDTC compared to the induction-only group (*p* > 0.05).

## 4. Discussion

The present in vitro study sheds light on the effects of cordycepin administration on DPSC and the influence of the bioactive molecule on osteogenic and adipogenic differentiation of the DPSC. It is well documented that DPSC derived from the human dental pulp are a good source and niche of stem cells in the human body. These cells of mesenchymal origin have been exploited for their multipotent nature and plasticity to regenerate lost organs and tissue parts in patients with debilitating and mutilating diseases [13]. The primary advantage of using DPSC is the ease associated with the process of harvesting these cells from the patients as the accessibility of the cells is good [4]. Moreover, the process of obtaining human dental pulp is not as invasive as obtaining bone marrow tissue and is not associated with any significant morbidity.

In the present study, the DPSC were obtained from a reputed molecular biology lab from their stem cell bank. However, to check the authenticity and characteristics, the DPSC were subjected to flow cytometry procedures to assess the levels of various surface markers. The results revealed a high expression of CD 73, CD 90, and CD and faint expression of CD 34, CD 45, and HLA-DR These findings reiterate the fact that the cells obtained from the laboratory have all the characteristic features of DPSC. These findings are concurrent with those of published literature that have assessed potential surface markers of DPSC and have found similar expression profiles [14].

After the characterization step, the DPSC was used for an MTT assay following administration of various concentrations of cordycepin. Data based on the assay revealed that 5 µM cordycepin was an ideal concentration to carry out further studies. Hence, this concentration was used for all the further studies. Experiments assessing adipogenesis and osteogenesis-related genes were assessed in the DPSC following cordycepin treatment with and without the use of induction factors.

Concerning the experiments assessing adipogenesis related genes in the DPSC following induction and cordycepin treatment, it was found that there was a statistically significant lowering of all four adipogenesis related genes PPARγ, FABP4, LPL, and C/EBPα, following cordycepin treatment in the presence of induction compared to the only induction group and untreated control cells. These results highlight the fact that cordycepin is an inhibitor of adipogenesis. This finding has already been proven by a previous study that has documented that cordycepin inhibits adipogenesis and the differentiation of preadipocytes into adipocytes [15,16,17]. Similar to this present study, Takahashi et al., 2012 have reported that cordycepin causes blockade of mTORC1 via inhibition of PKB (Akt) and activation of AMP kinase thereby inhibiting the adipogenic pathway. Early induction of the adipogenic C/EBPβ-PPARγ pathway was suppressed by cordycepin in the above-mentioned study [15]. Thus it can be inferred that the decreased expression of PPARγ and C/EBPα in the cells treated with cordycepin could be attributed to the suppression of C/EBPβ-PPARγ pathway by cordycepin. 

Concerning the experiments assessing osteogenesis properties of DPSC following cordycepin administration, it was found that there was a statistically significant elevation in osteogenesis-related genes along with a subsequent increase in ALP activity and alizarin red staining in the DPSC subjected to cordycepin. With regard to factors that promote mineralization potential in DPSCs, NF-κB deserves mention as it is a potent transcription factor that can switch on BMP2, ALP, COL1A1, and RUNX2 genes that enhance mineralization. We hence chose PDTC, a pharmacologic inhibitor of NF-κB to intentionally retard the genes that enhance mineralization and to check if cordycepin could rescue these inhibitory changes. The interesting fact was that upon induction and subsequent addition of an NF-κB inhibitor and cordycepin, there was still an increase in the osteogenic potential of the DPSC. These findings go to prove the osteopromotive activities of cordycepin. The bone sparing properties of cordycepin have been observed in mesenchymal stem cells derived from the human bone marrow where similar findings like those of the present study have been observed [18]. It has been documented that hypoxia is an important phenomenon that promotes osteogenesis and osteogenic differentiation of stem cells. By its beneficial effects, cordycepin has been found to cause hypoxia thereby exerting antioxidant effects and preventing excessive oxidative stress [18]. Another significant molecular effect of cordycepin is its inhibitory effect on TNF alpha, a proinflammatory cytokine. By this mechanism, cordycepin can cause indirect inhibition of NF kappa B and thereby restores osteogenesis. Cordycepin is also known to exert widespread anti-inflammatory effects by its effects on the NF-κB, RIP2/Caspase-1, Akt/GSK-3β/p70S6K, TGF-β/Smads, and Nrf2/HO-1 signaling pathways [19]. Thus, the immunomodulatory effect of cordyepin could also play a vital role in induction of osteogenesis.

The preliminary insights given by the present study highlight the positive effects on osteogenic differentiation of DPSCs. This study is novel as there are no studies performed similarly in the past. Medicinal mushrooms have been cultivated in various parts of the globe and can produce thousands of phytoactive compounds [20], one of them being cordycepin. It has been documented that these mushrooms are easy to culture, and the process of fermentation for cordycepin isolation is also easy [19]. With the advances in medicinal chemistry chemical synthesis of bioactive compounds is also being performed easily with successful results. The limitations of the study include that the study has an in vitro design and has been performed only on DPSCs and has not been tested other stem cells such as stem cells from human exfoliated deciduous teeth (SHED) and gingival mesenchymal stem cells (GMSCs). It is noteworthy that beta-actin gene expression varies widely in stem cells of mesenchymal origin and widely varies in intensity as the cells differentiate into various lineages. This fact could be considered a potential limitation of the study as we assessed the differentiation of the DPSCs into two different lineages. Another limitation of the study is based on the fact that we have not reported the adherence of the PCR technique to the MIQE guidelines [20]. This limitation can also be justified in part as the present study is not an entirely PCR based study and has followed many other molecular biology techniques. However, we plan to perform future studies with enhanced standardization protocols. Additionally, the results of the present study have to be extrapolated clinically and the role of possible influence of external factors on the results of the study should also be analyzed. Hence, future studies in different conditions (such as variations in oxygen levels, temperature, stages of cellular inflammation), studies in different dental stem cells (such as periodontal ligament derived stem cells, PDLSCs, SHED) can be performed. The need of the hour is to devise the methods by which cordycepin can be delivered onto sites where DPSC is being used for attempting regeneration. Moreover, the interaction of this compound with biomaterials and scaffolds needs to be understood before applying it for clinical use. Animal studies are needed before taking cordycepin for testing on humans. If fruitful results are obtained cordycepin could be used as a wonder drug to regenerate bone using DPSCs and could be a boon to regenerative medicine.

## 5. Conclusions

The present study was performed to assess the in vitro adipogenic and osteogenic differentiation potential of human dental pulp stem cells with or without induction after administration of cordycepin. We observed that cells treated with optimum concentration of cordycepin (5 µM) caused a significant decrease in expression of the tested genes related to adipogenesis, namely PPARγ, FABP4, LPL, and C/EBPα. Considering osteogenesis, cordycepin caused a significant increase in the expression of RUNX2, COL1A1, OSX and OCN genes along with the increase in Alkaline phosphatase and alizarin red staining in treated DPSCs along with the presence of induction and simultaneous addition of PDTC. Thus cordycepin could be explored further for its osteopromotive properties and could be used as a bioactive molecule alongside the administration of dental pulp stem cells in the field of regenerative medicine.

## Figures and Tables

**Table 1:** List of primers.

Gene	Forward Primer	Reverse Primer
*PPARγ*	5′-AGC CTG CGA AAG CCT TTT GGT G-3′	5′-GGC TTC ACA TTC AGC AAA CCT GG-3′
*FABP4*	5′-ACG AGA GGA TGA TAA ACT GGT GG-3′	5′-GCG AAC TTC AGT CCA GGT CAA C-3′
*LPL*	5′-CTG CTG GCA TTG CAG GAA GTC T-3′	5′-CAT CAG GAG AAA GAC GAC TCG G-3′
*CEBPα*	5′-AGG AGG ATG AAG CCA AGC AGC T-3′	5′-AGT GCG CGA TCT GGA ACT GCA G-3′
*RUNX2*	5′-GTG CCT AGG CGC ATT TCA-3′	5′-GCT CTT CTT ACT GAG AGT GGA AGG-3′
*COL1A1*	5′-GAT TCC CTG GAC CTA AAG GTG C-3′	5′-AGC CTC TCC ATC TTT GCC AGC A-3′
*OSX*	5′-TGC TTG AGG AGG AAG TTC AC-3′	5′-AGG TCA CTG CCC ACA GAG TA-3′
*OCN*	5′-GGC GCT ACC TGT ATC AAT GG-3′	5′-TCA GCC AAC TCG TCA CAG TC-3′
*ACTB*	5′-AGA GCT ACG AGC TGC CTG AC-3′	5′-AGC ACT GTG TTG GCG TAC AG-3′

**Table 2:** Flow cytometry analysis of MSC-specific markers in DPSCs.

MSC Markers	Percentage Positive Cells
CD73	98.0%
CD90	99.0%
CD105	96.7%
CD34	0.6%
CD45	1.3%
HLA-DR	0.2%

**Table 3:** MTT assay at 48 h following cordycepin treatment of DPSC.

Concentration	MTT—48 h Absorbance at 570 nm
Control	0.58 ± 0.02
0.5 µM Cpn	0.62 ± 0.12
1 µM Cpn	0.59 ± 0.12
2.5 µM Cpn	0.64 ± 0.14
5 µM Cpn	0.71 ± 0.11
10 µM Cpn	0.43 ± 0.13
25 µM Cpn	0.27 ± 0.06
50 µM Cpn	0.19 ± 0.04

**Table 4:** Relative expression of adipogenesis related genes in the DPSC following treatment with cordycepin.

Gene	Control	ADIP-Induced	Cordycepin + ADIP-Induced
PPARγ	0.00014 ± 0.000037	0.0085 ± 0.0017	0.0010 ± 0.00019
FABP4	0.000041 ± 0.000011	0.00059 ± 0.00012	0.000082 ± 0.000016
LPL	0.000063 ± 0.000016	0.00083 ± 0.00017	0.00032 ± 0.000063
C/EBPα	0.00020 ± 0.000054	0.0013 ± 0.00026	0.00042 ± 0.000082

**Table 5:** Relative expression of osteogenesis related genes in the DPSC following treatment with cordycepin.

Gene	Control	OST-Induced	PDTC + OST-Induced	PDTC + Cordycepin + OST-Induced
RUNX2	0.00032 ± 0.000087	0.0047 ± 0.00098	0.00021 ± 0.000041	0.0051 ± 0.0011
COL1A1	0.0034 ± 0.00092	0.066 ± 0.013	0.0042 ± 0.00082	0.074 ± 0.016
OSX	0.00018 ± 0.000050	0.0077 ± 0.0015	0.00010 ± 0.000019	0.0085 ± 0.0018
OCN	0.00034 ± 0.000092	0.0012 ± 0.00026	0.00019 ± 0.000037	0.0013 ± 0.00029

**Table 6:** Quantification of mineralization alizarin red staining—day 21.

Concentration	Mineralization (Absorbance at 450 nm)
Control	0.13 ± 0.03
OST-Induced	2.13 ± 0.43
PDTC + OST-induced	0.21 ± 0.04
PDTC + cordycepin + OST-induced	2.82 ± 0.61

**Table 7:** Quantification of ALP activity—day 21.

Concentration	ALP Activity (Absorbance at 405 nm)
Control	0.35 ± 0.09
OST-Induced	1.12 ± 0.22
PDTC + OST-induced	0.11 ± 0.022
PDTC + cordycepin + OST-induced	1.69 ± 0.36

## Abbreviations

DPSCs	Dental pulp stem cells
FBS	Fetal bovine serum
MTT	3-(4,5-dimethylthiazol-2-yl)-2,5-diphenyltetrazolium bromide
Cpn	Cordycepin
DMSO	dimethyl sulfoxide
PDTC	Pyrrolidine dithiocarbamate
DMEM	Dulbeco’s Modified Eagle Medium
EDTA	Ethylenediaminetetraacetic acid
ALP	Alkaline phosphatase
NF	nuclear factor (NF)
RUNX2	Runt-related transcription factor 2
COL1A1	Collagen, type I, alpha 1
OSX	Osterix
OCN	Osteocalcin
